# Associations of iron metabolism genes with blood manganese levels: a population-based study with validation data from animal models

**DOI:** 10.1186/1476-069X-10-97

**Published:** 2011-11-10

**Authors:** Birgit Claus Henn, Jonghan Kim, Marianne Wessling-Resnick, Martha María Téllez-Rojo, Innocent Jayawardene, Adrienne S Ettinger, Mauricio Hernández-Avila, Joel Schwartz, David C Christiani, Howard Hu, Robert O Wright

**Affiliations:** 1 Department of Environmental Health, Harvard School of Public Health, Boston, MA, USA; 2 Department of Genetics and Complex Diseases, Harvard School of Public Health, Boston, MA, USA; 3 Division of Statistics, Center for Evaluation Research and Surveys, National Institute of Public Health, Cuernavaca, Morelos, Mexico; 4 Channing Laboratory, Department of Medicine, Brigham and Women's Hospital, Harvard Medical School, Boston MA, USA; 5 Center for Perinatal, Pediatric and Environmental Epidemiology, Department of Epidemiology and Public Health, Yale University School of Medicine, New Haven, Connecticut 06510, USA; 6 Ministry of Health, Mexico City, Mexico; 7 Department of Environmental Health Sciences, University of Michigan School of Public Health, Ann Arbor, MI, USA; 8 Department of Emergency Medicine, Children's Hospital Boston, Boston, MA, USA

**Keywords:** Iron, Manganese, Genes, Iron metabolism genes

## Abstract

**Background:**

Given mounting evidence for adverse effects from excess manganese exposure, it is critical to understand host factors, such as genetics, that affect manganese metabolism.

**Methods:**

Archived blood samples, collected from 332 Mexican women at delivery, were analyzed for manganese. We evaluated associations of manganese with functional variants in three candidate iron metabolism genes: *HFE *[hemochromatosis], *TF *[transferrin], and *ALAD *[δ-aminolevulinic acid dehydratase]. We used a knockout mouse model to parallel our significant results as a novel method of validating the observed associations between genotype and blood manganese in our epidemiologic data.

**Results:**

Percentage of participants carrying at least one copy of *HFE C282Y*, *HFE H63D*, *TF P570S*, and *ALAD K59N *variant alleles was 2.4%, 17.7%, 20.1%, and 6.4%, respectively. Percentage carrying at least one copy of either *C282Y *or *H63D *allele in *HFE *gene was 19.6%. Geometric mean (geometric standard deviation) manganese concentrations were 17.0 (1.5) μg/l. Women with any *HFE *variant allele had 12% lower blood manganese concentrations than women with no variant alleles (β = -0.12 [95% CI = -0.23 to -0.01]). *TF *and *ALAD *variants were not significant predictors of blood manganese. In animal models, *Hfe*^-/- ^mice displayed a significant reduction in blood manganese compared with *Hfe*^+/+ ^mice, replicating the altered manganese metabolism found in our human research.

**Conclusions:**

Our study suggests that genetic variants in iron metabolism genes may contribute to variability in manganese exposure by affecting manganese absorption, distribution, or excretion. Genetic background may be critical to consider in studies that rely on environmental manganese measurements.

## Background

Manganese (Mn) is an essential nutrient but also a known neurotoxicant at high chronic exposure levels. In particular, occupational studies have documented adverse effects on motor, psychological, and neurological function following long term exposures in adults, with progressively worse symptoms persisting long after cessation of exposure [1-6]. While most epidemiologic literature on manganese toxicity has focused on occupational exposure, there is emerging evidence that elevated environmental levels of manganese may cause cognitive and motor deficits, as well as behavioral problems, among both adults and children [7-15]. High maternal blood manganese levels have also been associated with lower birth weights, although this effect appears nonlinear [16].

Given the emerging evidence for adverse effects from excess manganese exposure, it is critical to understand host factors that affect manganese metabolism. Such factors may provide mechanistic clues for future interventions or may identify susceptible subpopulations. For example, there is a well-known inverse association between iron stores and manganese absorption. In laboratory animals, iron deficiency results in increased absorption and retention of manganese in various organs [17-21]. Iron overload, in contrast, reduces manganese accumulation in the brain and in other organs [20]. In humans, similar inverse associations of blood manganese with ferritin and dietary intake of nonheme iron have been reported [22-24]. It is therefore likely that iron and manganese share the same transport and regulatory proteins, and at least one factor, Divalent Metal Transporter-1 (DMT1), is known to mediate uptake of both cations [25].

Manganese and iron are both transition metals and, given their similar chemical properties, likely share genetic factors that regulate their metabolism. Such genetic factors may alter manganese absorption, excretion, or tissue/cellular distribution, thereby altering blood manganese levels. Variants of iron metabolism genes with known functional properties, such as the *HFE *gene, may be good candidates to regulate manganese metabolism. The *HFE *gene product regulates levels of the regulatory hormone hepcidin, thereby controlling iron absorption in response to body iron status. Two well-characterized, functional missense variants in the *HFE *gene, *C282Y *and *H63D*, have been associated with increased dietary iron uptake [26,27]. The *C282Y *variant is the primary cause of the common form of hereditary hemochromatosis, a genetic disease of excess iron absorption. Transferrin is an iron transport protein that delivers iron from the gastrointestinal tract to tissues. A common missense variant in the *TF *(transferrin) gene, *P570S *(or *TFC2*), has been associated with differential binding of iron to transferrin relative to the wildtype protein [28]. Finally, *ALAD *(δ-aminolevulinic acid dehydratase) is part of the heme synthesis pathway [29]. Heme is a necessary component of hemoglobin and all cytochrome enzymes, and requires iron for enzyme activity. This iron metabolism gene has been linked to metal toxicity [30-32] as well as metal binding [33]. The *K59N *(or *ALAD2*) variant of this gene has also been associated with increased hemoglobin [34]. We hypothesized that, because each of these iron metabolism genes has common functional variants and because iron metabolism is linked to manganese metabolism, these variants may be associated with altered blood manganese levels. We also explored whether any observed effects of genotype are mediated through iron status.

We examined these questions using blood manganese levels in a cohort of women at time of delivery. Pregnancy is a time at which blood manganese levels increase [35,36] as well as a time of relative iron deficiency. We believed the increase in variability of both iron stores and blood manganese would increase our ability to detect differences in blood manganese related to genetic variants in iron metabolism genes. This time point is also relevant because maternal blood manganese is a measure of prenatal exposure to the fetus, and there is evidence that environmental manganese exposure in utero is associated with later life behavioral disinhibition [9] and lower psychomotor development test scores among offspring [12]. Cost constraints prevented a large scale or full interrogation of the iron metabolism gene pathway. Instead, we focused specifically on three candidate iron metabolism genes, *HFE*, *TF*, and *ALAD*, which have either known or putatively functional variants. In order to validate that our findings were not due to chance or linkage disequilibrium (LD) with a nearby functional variant, we used a knockout mouse model to determine if we could replicate our results in an experimental setting. This is a novel method of validating observed associations involving genetics and observational epidemiologic data. It is not subject to population substructure and is more cost effective than replicating findings in an independent population. To our knowledge, this is also the first study to examine blood manganese levels in HFE-deficient mice.

## Methods

### Study participants

The human subjects committees of the National Institute of Public Health of Mexico, Harvard School of Public Health, and participating hospitals approved all study materials and procedures. Study participants were identified from among pregnant women who were eligible to participate in a randomized trial of calcium supplementation to lower blood lead levels among postpartum lactating women [37]. Women were recruited for this trial between January 1994 and June 1995 from three maternity hospitals in Mexico City that serve a low- to moderate-income population. Data collection and exclusion criteria are detailed elsewhere [38]. Briefly, women were excluded due to: factors that could interfere with maternal calcium metabolism; logistic reasons that would interfere with data collection; intention not to breastfeed; and certain medical conditions.

Demographic and social characteristics were collected from women within 12 hours of delivery. For the trial, venous blood samples were collected for lead measurement, genotyping, and iron status (i.e., serum ferritin and hemoglobin, when possible) at or near the time of delivery. From a total of 614 women eligible for randomization, 571 were successfully genotyped (4 samples were excluded due to blood data collection errors). The availability of blood for DNA extraction was given priority; therefore, the blood sample with the higher volume was chosen for DNA extraction/genotyping. Any remaining blood was archived and subsequently analyzed for manganese for use in the present analysis. Three hundred thirty-two (54 percent) had archived blood samples from delivery still available for measurement of manganese concentration. Manganese does not degrade; therefore, our results should not be affected by storage. Because we measured blood manganese at time of delivery, our data predate the calcium intervention trial and the randomization process cannot affect our results. At 1-month postpartum, additional blood samples were collected for hemoglobin and ferritin measurement.

### Genotyping

High-molecular-weight DNA was extracted from white blood cells with commercially available PureGene Kits (Gentra Systems, Minneapolis, MN). After DNA quantification, samples were adjusted to TE buffer, partitioned into aliquots, and stored at -80°C. To design multiplex PCR assays, we used Sequenom SpectroDESIGNER software and inputted sequence containing the SNP and 100 bp of flanking sequence on either side of the SNP. Four SNPs were multiplexed: (*HFE*) *C282Y *(rs1800562) and (*HFE*) *H63D *(rs1799945), transferrin (*TF*) *P570S *(rs1049296), and δ-aminolevulinic acid dehydratase (*ALAD*) *K59N *(rs1800435). The extension product was then spotted onto a 384-well spectroCHIP before being flown in the MALDI-TOF mass spectrometer. Reference sequences and primers used in the multiplex assay are provided in Additional file 1.

### Measurement of blood manganese

Blood samples were collected in trace metal-free tubes at delivery from all participants and immediately frozen. Because blood manganese levels change over the course of pregnancy [35,36], it was necessary to collect samples at approximately the same time point for all women. We chose samples from delivery, which also avoids any influence from the calcium trial. Blood samples were prepared and analyzed for manganese concentrations at the Trace Metals Laboratory at Harvard School of Public Health in Boston, MA. Metals concentrations were measured with a dynamic-reaction cell-inductively-coupled plasma mass spectrometer (DRC-ICP-MS, Elan 6100, Perkin Elmer, Norwalk, CT), using previously described methods and quality control measures [16]. In short, blood samples were weighed (1 g) and digested in HNO_3 _acid, followed by addition of H_2_O_2 _at room temperature, and then diluted with deionized water. Quality control measures included initial and continuous calibration verification standards, procedural blanks, duplicate samples, spiked samples, and the National Institute of Standards and Technology Standard Reference Material for trace elements in water (NIST SRM 1643d). Although iron may cause interference of the manganese measurement on ICP-MS, due to the high concentration of iron (^54^Fe) and its proximity to manganese (^55^Mn) on the periodic table, any potential interference is not expected to change the rankings of blood manganese levels in our samples. Recovery rates for manganese in quality control samples were 83%-100%, and precision (%RSD) was less than 10%. The average limit of detection was 0.09 μg/dl.

### Blood manganese in HFE-deficient mice

Animal protocols were approved by the Harvard Medical Area Animal Care and Use Committee. HFE-deficient (*Hfe*^-/-^) and "wildtype" control (129/SvEv; *Hfe*^+/+^) mice were kindly provided by Dr. Nancy Andrews (Duke University) to establish a breeding colony. Mice were maintained on a 12-h light/dark cycle and consumed a facility chow containing 220 mg/kg iron (PicoLab 5058, PharmaServ) and water ad libitum. The mutation involved in the *Hfe*^-/- ^phenotype was verified by PCR as described by Levy et al. [39]. At four months of age (young adult; considered to be comparable in age to participants in our human study), female mice were euthanized by isoflurane overdose followed by exsanguination for collection of the whole blood and liver. A separate cohort of female HFE-deficient and "wildtype" control mice (8-9 months old) was also examined for blood manganese levels 3-4 weeks postpartum, in order to examine whether pregnancy history affects the relationship between genotype and manganese level. Blood levels of manganese were determined by the Trace Metals Laboratory as described above. Blood iron concentrations were measured using Inductively Coupled Plasma Optical Emission Spectroscopy (ICP-OES) (Optima 7300, Perkin Elmer, Norwalk, CT) using yttrium (λ = 371.029 nm) as the internal standard. Iron concentration given was the average of the five replicate measurements at the wave length 238.204 nm. Liver non-heme iron was determined using the method of Torrance and Bothwell [40]. Briefly, mouse liver tissues (~200 mg) were incubated in 40-fold volume of acid solution (10% trichloroacetic acid, 3 M HCl) at 65°C for 20 hours. After centrifugation, supernatant was mixed with half-saturated sodium acetate containing thioglycolic acid (0.1%) and bathophenanthroline sulfonate (0.01%) and spectrophotometric assay was employed to determine the absorbance at 535 nm.

### Statistical analysis

In the human study, we tested the distribution of genotype frequencies using a chi-square statistic to compare observed and expected counts according to principles of Hardy-Weinberg equilibrium. Because of the relatively low allele frequencies, we chose dominant genetic models for each allele, combining heterozygotes with homozygote variants into a single indicator term for subsequent analyses. Although the *C282Y *variant allele appears to have a more severe phenotype with respect to iron metabolism than the *H63D *variant [41,42], we decided a priori to combine the two *HFE *alleles (*C282Y *and *H63D*) if effects were in the same direction for each variant, as we have done previously for blood/bone lead and *HFE *variants [43,44].

**Figure 1:**
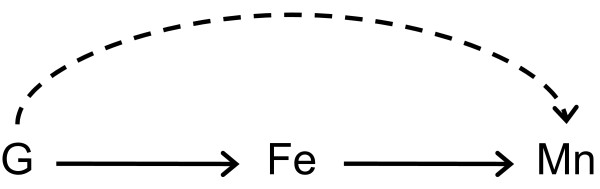
**Directed acyclic graph depicting potential pathways for iron metabolism genes to affect blood manganese levels**. Genotype, G, may cause changes to iron levels, Fe, which subsequently affect blood manganese levels, Mn (pathway represented by solid arrows). Alternatively, genotype, G, may act on blood manganese levels, Mn, through another pathway, independent of iron levels (pathway represented by dashed arrow).

Univariate summary statistics and distributional plots were examined for blood manganese and for covariates. As residuals of blood manganese concentrations were distributed log normally, we transformed manganese concentrations to their natural logarithm to achieve approximate normality. We calculated geometric means and geometric standard deviations (GSDs) of blood manganese concentrations for all study participants and stratified by genotype. Bivariate associations of covariates with blood manganese were also examined. Covariates considered were: age at time of delivery, marital status, education (number of years), number of pregnancies, type of delivery (vaginal or Caesarian section), newborn sex, gestational age of newborn (weeks), hemoglobin (at 1-month postpartum), and ferritin (at time of delivery and 1-month postpartum). We used linear regression to model the effects of genotypes on maternal blood manganese at delivery. Because manganese levels were natural log-transformed, beta coefficients in regression models represent a percent change in manganese levels for carriers of the variant genotype compared to wildtype subjects. We also hypothesized that any potential effects of genotypes on manganese are mediated through iron status (see Figure 1). To evaluate this, we fit all models adjusted for hemoglobin. We modeled hemoglobin as a linear variable because we observed no significant departures from linearity in the association between hemoglobin and manganese. We further adjusted models for covariates observed to be associated with blood manganese. We did not expect substantial confounding by these covariates because they are unlikely to predict genotype, a necessary condition for factors to be confounders. As the original study protocol did not include a complete blood count (CBC) at delivery, hemoglobin measured at 1-month postpartum was used as a proxy for hemoglobin at the time of delivery, due to the large number of missing data for this variable at delivery. All statistical analyses were performed using SAS version 9.1.3 (SAS Institute Inc., Cary, NC, USA).

For animal data, we used an unpaired Student's t-test to compare mean body weight, liver non-heme iron, blood iron, and blood manganese in HFE-deficient (*Hfe*^-/-^) and "wildtype" control (*Hfe*^+/+^) mice.

## Results

### Characteristics of study participants

A total of 332 women had available blood manganese data at delivery and were successfully genotyped. Characteristics of these women are similar to those of non-participating women (Table 1). Among participants, 2.4%, 17.7%, 20.1%, and 6.4% carried at least one copy of the *HFE C282Y*, *HFE H63D*, *TF P570S*, and *ALAD K59N *variant alleles, respectively. Also, 19.6% carried at least one copy of either the *C282Y *or *H63D *allele. Polymorphisms were in Hardy-Weinberg equilibrium (see Table 2).

**Table 1:** Characteristics of Study Participants and Nonparticipants

	Participants		Nonparticipants	
**Characteristics**	**N**	**Mean (SD) or %**	**N**	**Mean (SD) or %**

Age at delivery (years)	332	24.3 (5.2)	281	24.7 (5.1)

Marital status (% married)	332	65.1%	281	64.4%

Education (years)	325	9.2 (3.0)	279	9.5 (3.2)

Number of pregnancies	332	2.0 (1.3)	281	2.0 (1.2)

Type of delivery (% Caesarian section)	331	19.0%	277	21.3%

Newborn sex (% male)	332	53.0%	278	56.1%

Gestational age of newborn (weeks)	329	39.2 (1.4)	276	39.1 (1.6)

Measures of iron status:				

Hemoglobin^b ^(g/dl)	329	13.5 (1.5)	273	13.6 (1.4)

Ferritin^a ^(ng/ml)	124	22.3 (27.8)	65	21.4 (22.1)

Ferritin^b ^(ng/ml)	122	28.9 (33.9)	68	25.9 (24.0)

^a ^Collected at time of delivery

^b ^Collected at 1-month postpartum

**Table 2:** Genotype Frequencies for Study Participants (N = 332)

Genotype	N (%)
*HFE C282Y*^a^	

Homozygous Wildtype (CC)	322 (97.6)

Heterozygous (CY)	8 (2.4)

Homozygous Variant (YY)	0 (0)

*HFE H63D*^b^	

Homozygous Wildtype (HH)	270 (82.3)

Heterozygous (HD)	56 (17.1)

Homozygous Variant (DD)	2 (0.6)

*TF P570S*^c^	

Homozygous Wildtype (CC)	263 (79.9)

Heterozygous (CT)	65 (19.8)

Homozygous Variant (TT)	1 (0.3)

*ALAD K59N*^d^	

Homozygous Wildtype (GG)	309 (93.6)

Heterozygous (GC)	21 (6.4)

Homozygous Variant (CC)	0 (0)

^a ^N = 2 missing genotype data; results in Hardy-Weinberg equilibrium: X^2 ^= 0.05, p = 1.00

^b ^N = 4 missing genotype data; results in Hardy-Weinberg equilibrium: X^2 ^= 0.24, p = 1.00

^c ^N = 3 missing genotype data; results in Hardy-Weinberg equilibrium: X^2 ^= 2.11, p = 0.23

^d ^N = 2 missing genotype data; results in Hardy-Weinberg equilibrium: X^2 ^= 0.36, p = 1.00

### Blood manganese concentrations in study participants

Blood manganese concentrations for all participants and stratified by genotype are presented in Table 3. Among all participants, manganese concentrations ranged from 4.2 to 66.2 μg/L, with a geometric mean (GSD) of 17.0 (1.5) μg/L (Table 3). Fifth and 95^th ^percentiles were 8.7 and 32.8 μg/L. Compared to wildtype *HFE *carriers, geometric mean manganese was lower among carriers of *HFE *variants (any *HFE *variant: 15.4 (1.5) μg/L vs. wildtype: 17.4 (1.5) μg/L, p = 0.04). Carriers of *TF *and *ALAD *variants had similar manganese concentrations as wildtypes.

**Table 3:** Blood Manganese (μg/L) for All Study Participants and by Genotype

	N	GM^a ^(GSD^b^)
All	332	17.0 (1.5)

*HFE C282Y*		

Wildtype	322	17.1 (1.5)

Variant	8	13.5 (1.4)

*HFE H63D*		

Wildtype	270	17.2 (1.5)

Variant	58	15.8 (1.5)

*HFE *(*C282Y *or *H63D*)		

Wildtype	266	17.4 (1.5)

Variant	65	15.4 (1.5)

*TF P570S*		

Wildtype	263	16.9 (1.5)

Variant	66	17.1 (1.4)

*ALAD K59N*		

Wildtype	309	16.9 (1.5)

Variant	21	17.3 (1.5)

^a ^GM = geometric mean

^b ^GSD = geometric standard deviation

### Associations between genotype, covariates, and manganese concentrations

Table 4 summarizes associations between covariates and log-transformed blood manganese concentrations. Both hemoglobin and ferritin were inversely associated with blood manganese, though only ferritin at time of delivery was statistically significant (β = -0.002, 95% CI: -0.005 to -0.0001). This inverse association between iron and manganese is consistent with other animal and human studies [17,20,23], as described earlier. Gestational age was also inversely associated with blood manganese (β = -0.03, 95% CI: -0.06 to -0.002).

**Table 4:** Crude Associations of Covariates with Log-transformed^a ^Blood Manganese

Covariates	N	Beta	(95% CI)
Age at delivery (years)	332	-0.002	(-0.01 to 0.01)

Marital Status:			

Living with partner (vs. married)	332	0.04	(-0.07 to 0.14)

Separated/divorced (vs. married)		-0.05	(-0.21 to 0.12)

Education (years)	325	-0.003	(-0.02 to 0.01)

Number of pregnancies	332	-0.02	(-0.05 to 0.02)

Type of delivery (Caesarian section vs. vaginal)	331	0.02	(-0.09 to 0.13)

Newborn sex (female vs. male)	332	0.05	(-0.03 to 0.14)

Gestational age of newborn at birth (weeks)	329	-0.03	(-0.06 to -0.002)

Measures of iron status:			

Hemoglobin^c ^(g/dl)	329	-0.03	(-0.05 to 0.003)

Ferritin^b ^(ng/ml)	124	-0.002	(-0.005 to -0.0001)

Ferritin^c ^(ng/ml)	122	-0.001	(-0.003 to 0.001)

^a ^Blood manganese concentrations are transformed to their natural logarithms; beta coefficients therefore represent percent change in manganese

^b ^Collected at time of delivery

^c ^Collected at 1-month postpartum

Results from linear regression models of genotype predicting blood manganese concentrations are presented in Table 5. Women with any *HFE *variant allele had significantly lower blood manganese concentrations than women with no variant alleles (β = -0.12, 95% CI: -0.23 to -0.01). This association did not change when we adjusted for hemoglobin, and changed minimally when we additionally adjusted for gestational age (β = -0.11, 95% CI: -0.23 to -0.003). *TF *and *ALAD *genotypes were not significant predictors of blood manganese levels.

**Table 5:** Regression Models of Genotypes^a ^Predicting Log-transformed^b ^Blood Manganese (μg/L)

	Crude			Adjusted for Hemoglobin			Fully Adjusted^c^		
**Genotype**	**N**	**Beta**	**(95% CI)**	**N**	**Beta**	**(95% CI)**	**N**	**Beta**	**(95% CI)**

*HFE C282Y*	330	-0.23	(-0.52 to 0.05)	327	-0.23	(-0.51 to 0.06)	324	-0.22	(-0.53 to 0.08)

*HFE H63D*	328	-0.09	(-0.20 to 0.03)	325	-0.08	(-0.20 to 0.03)	322	-0.08	(-0.20 to 0.03)

*HFE*^d^	331	-0.12	(-0.23 to -0.01)	328	-0.12	(-0.23 to -0.01)	325	-0.11	(-0.23 to -0.003)

*TF P570S*	329	0.01	(-0.10 to 0.12)	326	0.02	(-0.09 to 0.13)	323	0.02	(-0.10 to 0.13)

*ALAD K59N*	330	0.02	(-0.16 to 0.20)	327	-0.01	(-0.20 to 0.18)	324	-0.02	(-0.20 to 0.17)

^a ^All models compare blood manganese levels among women with any variant allele to levels among women with no variant allele

^b ^Blood manganese concentrations are transformed to their natural logarithms; beta coefficients therefore represent percent change in manganese for carriers of variant alleles compared to women with no variant allele

^c ^Models adjusted for hemoglobin at 1-month postpartum and gestational age

^d ^*C282Y *or *H63D*

We also evaluated associations between genotypes and iron variables. No statistically significant associations were observed between any of the four SNPs and either hemoglobin or ferritin (Table 6).

**Table 6:** Associations of Genotypes^a ^with Measures of Iron Status

	Hemoglobin (1-month postpartum)			Ferritin (delivery)			Ferritin (1-month postpartum)		
**Genotype**	**N**	**Beta**	**(95% CI)**	**N**	**Beta**	**(95% CI)**	**N**	**Beta**	**(95% CI)**

*HFE C282Y*	327	0.15	(-0.94 to 1.24)	124	4.78	(-27.54 to 37.09)	122	-9.16	(-43.43 to 25.10)

*HFE H63D*	325	0.02	(-0.42 to 0.47)	123	3.52	(-9.77 to 16.81)	121	13.15	(-3.27 to 29.56)

*HFE*^d^	328	-0.003	(-0.42 to 0.42)	124	4.44	(-8.31 to 17.20)	122	10.83	(-4.66 to 26.33)

*TF P570S*	326	0.28	(-0.15 to 0.70)	123	-7.38	(-19.53 to 4.77)	121	0.38	(-15.31 to 16.08)

*ALAD K59N*	327	-0.39	(-1.09 to 0.30)	124	-16.29	(-39.26 to 6.67)	122	-12.78	(-43.51 to 17.94)

^a ^All models compare hemoglobin or ferritin levels among women with any variant allele to levels among women with no variant allele

^b ^*C282Y *or *H63D*

### Blood manganese concentrations in Hfe^-/- ^mice

Table 7 summarizes physiological parameters of 4-month-old female *Hfe*^-/- ^mice. While body weight did not differ between *Hfe*^-/- ^and *Hfe*^+/+ ^mice, liver non-heme iron was significantly elevated in the absence of the murine HFE gene, confirming the state of iron overload in *Hfe*^-/- ^mice, as previously reported by Levy et al. [39]. Blood iron levels were also higher in *Hfe*^-/- ^mice compared with *Hfe*^+/+ ^mice. In contrast, *Hfe*^-/- ^mice displayed a significant reduction in blood manganese level by ~30% compared with *Hfe*^+/+ ^mice (11.5 ± 0.8 ng/g vs. 15.8 ± 0.8 ng/g for *Hfe*^-/- ^and *Hfe*^+/+ ^mice, respectively), demonstrating impaired manganese homeostasis due to HFE deficiency. Since these mice had no pregnant history, we examined the blood manganese levels of a separate cohort of 8- to 9-month-old female *Hfe*^-/- ^and *Hfe*^+/+ ^control mice 3-4 weeks postpartum. The results showed a similar pattern: mean blood manganese was lower among *Hfe*^-/- ^mice compared with *Hfe*^+/+ ^mice (6.0 ± 1.8 ng/g vs. 8.7 ± 2.1 ng/g for *Hfe*^-/- ^and *Hfe*^+/+ ^mice, respectively; n = 5 per genotype; p = 0.06), suggesting that blood manganese levels are lower in HFE-deficient mice regardless of age and pregnancy history.

**Table 7:** Physiological Parameters of *Hfe*^+/^^+ ^and *Hfe*^-/^^- ^mice^a^

	*Hfe*^+/+^			*Hfe*^-/-^		
	**N**	**Mean**	**(SD)**	**N**	**Mean**	**(SD)**

Body weight (g)	4	23.4	(1.4)	4	22.9	(2.1)

Liver non-heme iron (μg/g tissue)	4	234.4	(26.1)	4	861.5*	(42.0)

Blood iron (μg/g tissue)	4	429.5	(7.0)	4	477.9*	(19.8)

Blood manganese (ng/g tissue)	4	15.8	(0.8)	4	11.5*	(0.8)

^a ^Four-month old female mice

* Significantly different between the two genotypes as determined by unpaired Student's *t*-test (*p *< 0.05)

## Discussion

Blood manganese levels in our study are similar to levels observed among women around the time of delivery in other studies. Most similar are concentrations among a cohort residing in Quebec, Canada (GM = 16 μg/L; 5^th^, 95^th ^percentiles: 10 μg/L, 26 μg/L)[45]. At 34-weeks gestation, the mean blood manganese level among a group of Australian women was 13 μg/L (SD = 4 μg/L) (i.e., mean (SD) = 230 (68) nmol/L)[35]. Higher manganese concentrations were reported in a mother-infant cohort living near a Superfund site in northeast Oklahoma (median = 22 μg/L; 5^th^, 95^th ^percentiles: 13 μg/L, 41 μg/L)[16], and among groups of women in Montreal, Canada (mean = 23 μg/L; 5^th^, 95^th ^percentiles: 6 μg/L, 52 μg/L)[46], and Paris, France (mean = 23 μg/L; 5^th^, 95^th ^percentiles: 12 μg/L, 40 μg/L [46]; and GM = 20 μg/L; 5th, 95th percentiles: 11 μg/L, 40 μg/L [12]). The differences in blood manganese levels between studies may be due to differences in analysis method (e.g., Takser et al. 2003 and 2004, Smargiassi et al. 2002, and Spencer 1999 used atomic absorption spectrometry, while Zota et al. 2009 used ICP-MS), but may also be due to differences in environmental exposure levels or to differences in a factor related to manganese absorption/metabolism (e.g., iron status).

We found that carriers of the *HFE *variant genotype had 12% lower blood manganese levels than wildtype subjects. *TF *and *ALAD *genotypes were not associated with manganese levels in our data. In our experimental animal study, our findings of reduced blood manganese among *Hfe^-/- ^*mice compared to "wildtype" control mice recapitulated the findings of our human population study and affirm the relationship observed between deficiency of *HFE *function and reduced blood manganese. The results from the animal study support the hypothesis that *HFE *genotype is the cause of the lower blood manganese levels in the epidemiologic study. This step is critical as observational epidemiologic studies may be confounded by ethnicity (population stratification) or by linkage disequilibrium with a nearby functional variant in a different gene (i.e. the measured genotype is not truly causative). The animal study cannot be subject to population stratification. While a knockout model cannot confirm that the results are not due to a variant within the *HFE *gene in LD with the *H63D/C282Y *variants, it does confirm that LD with a variant outside the *HFE *gene is not driving the results.

Few studies have examined associations between iron metabolism genes and biomarkers of manganese, and results are inconsistent. Contrary to our findings, Nichols and Bacon reported that hereditary hemochromatosis patients (i.e., patients with clinical disease) accumulate manganese [47]. However, given that this study was conducted in patients with known hemochromatosis, it is not analogous to our report. In a pilot study of 141 Ohio residents, Haynes et al. observed no significant associations of *HFE *and *TF *with hair or blood manganese [48]. The relationship between ambient air manganese and hair manganese, however, was significant after adjusting for *HFE *and *TF *genotypes, suggesting that manganese absorption varies by genotype. In a separate analysis of the same cohort as the present study, we observed lower hair manganese levels among one-month-old infants whose mothers carried the *HFE *variant genotype (mean (SD) = 1.8 (1.4) μg/g) compared to wildtype mothers (mean (SD) = 3.2 (4.4) μg/g) (natural log-transformed hair manganese: β = -0.5, 95% CI: -0.9 to -0.1), which is again consistent with results presented here for maternal blood manganese.

As in previous animal and human studies [17,20,23], ferritin was inversely associated with blood manganese, although an association between iron metabolism genes and hemoglobin or ferritin was not observed. One explanation for the lack of an observed association might be the relative insensitivity of hemoglobin and ferritin to iron status. In the clinical setting, measuring body iron status accurately is notoriously difficult, and the limitations of serum ferritin and whole blood hemoglobin are well known. Bone marrow iron concentration is considered the gold standard, but is seldom used due to the invasive nature of this measure. In *HFE*-associated hemochromatosis patients, increased transferrin saturation typically precedes elevated ferritin levels and may therefore provide a more accurate representation of iron stores in these individuals than ferritin [49]. If the mechanism by which *HFE *genotype is associated with reduced blood manganese does reflect a change in iron status, transport mechanisms affecting both metals could be involved and reduced manganese levels may reflect altered uptake and distribution due to excess iron stores competing for manganese binding and transport. Loss of *HFE *function is associated with abnormally low levels of the iron regulatory hormone hepcidin, which result in up-regulation of the basolateral iron exporter ferroportin and net dietary absorption of iron [50]. It remains uncertain whether ferroportin is involved in absorption of dietary manganese across the intestine. While increased ferroportin levels may enhance transfer of iron from the enterocyte to circulation, manganese export out of the enterocyte may be blocked. Alternatively, increased iron loading upon loss of *HFE *function may affect distribution of manganese between the blood and soft tissue compartments and/or may facilitate excretion of manganese via biliary secretion.

It is also conceivable that the *HFE *variant genotype might alter blood manganese levels independent of iron status. In Figure 1, a directed acyclic graph depicts two possible mechanisms by which iron metabolism genes may affect blood manganese concentrations: either via iron levels or independent of iron levels. The lack of association between genotype and iron variables in our human data suggests that *HFE *could impact manganese levels in a pathway independent of iron levels (i.e., via dashed arrow in Figure 1). Finally, we cannot exclude the possibility that our findings are due to another polymorphism in the *HFE *gene or in a proximal gene with which *HFE *variants are in LD. However, we note that *H63D *and *C282Y *are not in high LD and no single variant would be expected to be in LD with both *HFE *variants. The animal study results would mitigate the possible role of a variant with high LD outside the *HFE *gene driving the results. The physiological role of *HFE *in manganese metabolism can be explored in future animal experiments, and more closely examined in patient studies of individuals with *HFE*-associated hemochromatosis and other iron overload disorders.

### Limitations

The original study protocol did not include a CBC at delivery and hemoglobin was instead measured at 1-month postpartum. This was used as a proxy for hemoglobin at delivery. While hemoglobin levels at these two time points may be different, we believe that differences are likely to be similar among all women, and random with respect to genotype and blood manganese. Ferritin levels at delivery were positively, though weakly, correlated with hemoglobin (Pearson correlation = 0.17, p = 0.055) and ferritin (Pearson correlation = 0.43, p < 0.001) at 1-month postpartum. Furthermore, when we adjusted for serum ferritin at delivery among the 124 subjects with data available on this variable, the association between *HFE *genotype and blood manganese became slightly stronger (β = -0.21, 95% CI: -0.37 to -0.05). Among this subset of women with ferritin data at delivery, we also examined the crude and hemoglobin-adjusted associations between *HFE *genotype and blood manganese, and found very similar results to ferritin-adjusted associations (crude: β = -0.22, 95% CI: -0.38 to -0.06; hemoglobin-adjusted: β = -0.22, 95% CI: -0.38 to -0.06). It should be noted that ferritin and hemoglobin, peripheral biomarkers of body iron stores, are not the gold standard biomarkers of body iron. Bone marrow iron or liver biopsy, considered gold standards [51,52], were not available.

Our population consisted solely of Mexican women, who are likely an admixed population of Southern European and Native American populations. We were unable to measure or adjust for population stratification, and therefore, confounding by race/ethnicity may be a limitation of our study. Again, the animal results would confirm that population stratification did not drive our results for *HFE*, as the animal model would not be subject to confounding by ethnicity. We also chose genetic alleles with known functional effects plausibly related to manganese metabolism a priori. While this does not eliminate population stratification, it does reduce the probability that such confounding is driving our results. The probability that a gene knockout experiment would replicate findings in humans that were actually due to population stratification is remote.

The prevalence of the *C282Y *variant is low (2.4%) compared to populations in Europe (9.2%) and the Americas (9.0%)[53,54]. Our results are therefore largely driven by the *H63D *variant, which is more common in Mexico (prevalence = 17.7%). In Africa, India, and Australia, the *C282Y *prevalence rates are even lower (0-0.5%) than in Mexico [27]. Due to the wide variability in prevalence of the *C282Y *and *H63D *alleles among different populations, generalizability of our results may be limited and results may differ in different populations. Therefore, as in any genetic association study, replication in other populations is necessary.

Manganese levels increase during pregnancy until at least 34 weeks gestation [35], suggesting a vital role for manganese in fetal development, but appear to decrease with increasing age after birth [55-57]. The observed inverse association between blood manganese and gestational age among our participants who were mostly (99%) over 35 weeks gestation could reflect the start of a normal physiological decline in manganese levels in late pregnancy. Because blood manganese levels change over the course of pregnancy, we measured blood manganese at the time of delivery for all women. There may be concerns about misclassification of manganese levels, in part because the length of gestation was not the same for all infants. However, we believe that any misclassification of manganese levels is likely to be non-differential with respect to genotype.

Only 54% of women who participated in the original randomized trial had genotype data and sufficient blood at delivery for subsequent manganese analysis. We do not expect selection bias to be a problem, however, because study participants were not aware of their genotype at the time of blood collection. Individuals were also unaware that extra blood would be used for manganese analysis, as manganese was not an exposure of interest at the time of blood collection. Additionally, we note that characteristics of participants were similar to those of non-participants. Finally, this reduced sample size limits the statistical power to detect small effects of variant alleles, which may have precluded us from observing an association for *TF *and *ALAD *variants.

## Conclusions

Adverse health effects from manganese exposure are variable, and have been described as a "continuum of dysfunction" [11]. Some of this variability may be due to differences in how individuals metabolize manganese. A given dose may be preferentially absorbed or excreted conferring different levels of susceptibility to an individual. Iron status is one factor that may affect manganese absorption. Likewise, genetic factors may influence susceptibility to manganese health effects. Because manganese is also a nutrient, both low and high levels of exposure may produce toxicity, further highlighting the importance of genes that regulate manganese metabolism. Our study suggests that genetics, possibly independent of iron stores, may contribute to variability in manganese exposure and perhaps also to manganese effects by affecting manganese absorption, distribution, or excretion.

In summary, we found that carriers of *HFE *variant alleles have lower blood manganese levels, which may be beneficial among individuals exposed to high levels of environmental manganese, or could have negative implications if dietary manganese intake is insufficiently low. Data from our complementary animal experiments strongly support the conclusion that loss of HFE function is associated with reduced blood manganese. This is among the first studies to observe an effect of genetics on human manganese biomarkers and the first, to our knowledge, that affirmed results in an animal model. If our findings are confirmed in other populations, genotype will be critical to consider in studies that rely on environmental manganese measurements.

## List of Abbreviations

*ALAD*:δ-aminolevulinic acid dehydratase gene; Fe:Iron; *HFE*:Hemochromatosis gene; LD:Linkage disequilibrium; Mn:Manganese; *TF*:Transferrin gene

## Competing interests

The authors declare that they have no competing interests.

## Authors' contributions

BCH conducted data analyses for the epidemiologic study and was responsible for manuscript preparation. JK and MWR were responsible for animal study, including interpretation of results and manuscript preparation. MTR participated in study coordination, data collection, and manuscript review. IJ performed laboratory analyses related to animal study. AE provided assistance with data management and interpretation, and contributed to manuscript revisions. JS provided statistical support and manuscript feedback. DC contributed to manuscript preparation and revisions. MHA, HH, and RW conceived of the study, and participated in its design and coordination. RW also assisted with manuscript preparation and revisions. All authors read and approved the final manuscript.

## Supplementary Material

Additional file 1 **Reference sequences and primers**. Reference sequences for the four SNPs, and primers used in the multiplex assay. Click here for file
