# Supplementary material for: Associations of iron metabolism genes with blood manganese levels: a population-based study with validation data from animal models
Source: Environ Health. 2011 Nov 10;10:97. doi: 10.1186/1476-069X-10-97 (PMC3248860; doi:10.1186/1476-069X-10-97)
Supplement: Additional file 1 — Reference sequences and primers. Reference sequences for the four SNPs, and primers used in the multiplex assay. [file 1476-069X-10-97-S1.PDF]

Additional file 1. Reference sequences and primers.

Reference sequences for the four SNPs are as follows: (*HFE*) *C282Y* (rs1800562) and (*HFE*) *H63D* (rs1799945): NM_139011; transferrin (*TF*) *P570S* (rs1049296): NM_001063; and δ-aminolevulinic acid dehydratase (*ALAD*) *K59N* (rs1800435): NG_008716. The following primers were used in the multiplex assay:

HFE rs1800562

Forward PCR primer 5'-ACGTTGGATGTACCCCAGATCACAATGAGG-3'

Reverse PCR primer 5'-ACGTTGGATGTGGATAACCTTGGCTGTACC-3'

Extension primer 5'-GAAGAGCAGAGATATACGT-3'

HFE rs1799945

Forward PCR primer 5'-ACGTTGGATGTCTACTGGAAACCCATGGAG-3'

Reverse PCR primer 5'-ACGTTGGATGTTGAAGCTTTGGGCTACGTG-3'

Extension primer 5'-GCTGTTCGTGTTCTATGAT-3'

TF rs1049296

Forward PCR primer 5'-ACGTTGGATGTGAGTTGCTGTGCCTTGATG-3'

Reverse PCR primer 5'-ACGTTGGATGATCTTTCCGTGTGACCACAG-3'

Extension primer 5'-CGCATACTCCTCCACAG-3'

ALAD rs1800435

Forward PCR primer 5'- ACGTTGGATGTTCAACCCCTCTACCCACAC-3'

Reverse PCR primer 5'- ACGTTGGATGAGATCAAGACACAGCGTAGG-3'

Extension primer 5'- CTCAGCATCTCTTCCAGCCG-3'
